# Low-Dose Endotoxin Induces Late Preconditioning, Increases Peroxynitrite Formation, and Activates STAT3 in the Rat Heart

**DOI:** 10.3390/molecules22030433

**Published:** 2017-03-08

**Authors:** Márton Pipicz, Gabriella F. Kocsis, László Sárváry-Arantes, Péter Bencsik, Zoltán V. Varga, Péter Ferdinandy, Tamás Csont

**Affiliations:** 1 Department of Biochemistry, Faculty of Medicine, University of Szeged, Dóm tér. 9., H-6720 Szeged, Hungary; pipicz.marton@med.u-szeged.hu (M.P.); gokf1@leicester.ac.uk (G.F.K.); sarvaryl@gmail.com (L.S-A.); bencsik.peter@med.u-szeged.hu (P.B.); varga.zoltan@med.semmelweis-univ.hu (Z.V.V.); peter.ferdinandy@pharmahungary.com (P.F.); 2 Pharmahungary 2000 Ltd., Dóm tér. 9., H-6720 Szeged, Hungary; 3 Department of Pharmacology and Pharmacotherapy, Semmelweis University, Nagyvárad tér. 4., H-1089 Budapest, Hungary

**Keywords:** oxidative stress, ONOO^−^, iNOS, XOR, SAFE pathway, cardioprotection

## Abstract

Administration of low-dose endotoxin (lipopolysaccharide, LPS) 24 h before a lethal ischemia induces pharmacological late preconditioning. The exact mechanism of this phenomenon is not clear. Here we aimed to investigate whether low-dose LPS exerts late effects on peroxynitrite formation and activation of Akt, Erk, and STAT3 in the heart. Male Wistar rats were injected with LPS (*S. typhimurium*; 0.5 mg/kg i.p.) or saline. Twenty-four hours later, hearts were isolated, perfused for 10 min, and then used for biochemical analyses. LPS pretreatment enhanced cardiac formation of the peroxynitrite marker 3-nitrotyrosine. LPS pretreatment also increased cardiac levels of the peroxynitrite precursor nitric oxide (NO) and superoxide. The activities of Ca^2+^-independent NO synthase and xanthine oxidoreductase increased in LPS-pretreated hearts. LPS pretreatment resulted in significantly enhanced phosphorylation of STAT3 and non-significantly increased phosphorylation of Akt without affecting the activation of Erk. In separate experiments, isolated working hearts were subjected to 30 min global ischemia and 20 min reperfusion. LPS pretreatment significantly improved ischemia-reperfusion-induced deterioration of cardiac function. We conclude that LPS pretreatment enhances cardiac peroxynitrite formation and activates STAT3 24 h later, which may contribute to LPS-induced late preconditioning.

## 1. Introduction

Cardiovascular diseases including myocardial infarction are the leading cause of death in western societies. Cardiac injury associated with myocardial infarction and subsequent reperfusion due to therapeutic restoration of blood supply (i.e., ischemia-reperfusion injury) includes cell death, life-threatening arrhythmias, and myocardial contractile dysfunction [1]. Preconditioning (PreC) is a well-known phenomenon applied before a lethal ischemia to protect the heart [2]. The protection is biphasic with an early phase (lasts for hours) and a late phase (starts 12 h after PreC stimuli and lasts for ~72 h) [3]. The latter lasts longer and protects against myocardial stunning as well, which makes the late phase of PreC clinically more relevant [4]. Late PreC can be elicited by a wide variety of non-pharmacological (e.g., ischemia-reperfusion, heat stress, rapid ventricular pacing, exercise) and pharmacological (e.g., endotoxin, cytokines, nitric oxide donors, opioids) stimuli [5,6]. Pharmacological PreC is a non-invasive way to confer cardioprotection, therefore it has a great preventive and therapeutic potential.

Administration of a low-dose endotoxin, lipopolysaccharide (LPS), 24 h before a test ischemia-reperfusion has been shown to improve post-ischemic cardiac functional recovery thereby exerting pharmacological PreC [7]. The exact mechanism of endotoxin-induced late PreC is not entirely clear. Nitric oxide (NO) has been implicated as a mediator, and inducible NO synthase (iNOS) has been identified as a major source of NO in endotoxin-induced late PreC in the heart [8,9,10]. Besides NO, indirect evidence suggests that superoxide (O_2_^•−^) may also be involved in endotoxin-induced late cardioprotection [7,11]; however, the cardiac level of O_2_^•−^ after low-dose LPS pretreatment has not been determined yet.

Furthermore, increasing evidence suggests that enhanced formation of cardiac peroxynitrite (ONOO^−^), the reaction product of NO and O_2_^•−^, plays a role in early [12,13] and late phase of ischemia-induced delayed PreC as well [14]. However, data is still lacking regarding the delayed effect of cardioprotective low-dose LPS on ONOO^−^ formation in the heart.

Cardioprotective signalling pathways are barely investigated in late PreC elicited by LPS. Reperfusion injury salvage kinase (RISK) and survival activating factor enhancement (SAFE) signalling are well-known cardioprotective pathways [15,16] and have been implicated in the mechanism of ischemic and certain-types of pharmacological late PreC [5,17]. In LPS-induced late PreC the activation of Akt, a protein kinase of RISK pathway was shown to play a role [18]; however, the potential implication of transcription factor STAT3—the key member of SAFE—has not yet been tested.

Therefore, here we investigated whether low-dose cardioprotective LPS has any delayed effect on ONOO^−^ formation in the heart. Furthermore, we tested the late effect of low-dose LPS treatment on the activation of cardiac RISK (Akt, Erk) and SAFE (STAT3) pathways.

## 2. Results

### 2.1. LPS Pretreatment Improves Post-Ischemic Cardiac Function and LDH Release: Evidence for Delayed Cardioprotection

**Figure 1:**
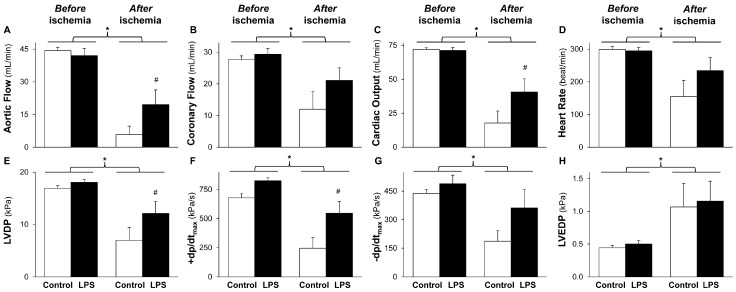
LPS pretreatment improves post-ischemic cardiac function. The figure shows cardiac functional parameters (**A**–**H**). Isolated rat hearts were subjected to 10 min equilibration period and 30 min normothermic global ischemia, followed by 20 min reperfusion, 24 h after in vivo 0.5 mg/kg low-dose lipopolysaccharide (LPS) treatment. Values are expressed as mean ± S.E.M (*n* = 6–7). * p < 0.05 vs before ischemia, *^#^ p* < 0.05 vs control, two-way ANOVA. LVDP: left ventricular developed pressure, ±d*p*/d*t*_max_: first derivatives of LVDP, LVEDP: left ventricular end-diastolic pressure.

Cardiac performance was measured in isolated hearts subjected to global ischemia 24 h after in vivo low-dose LPS (*S. typhimurium*; 0.5 mg/kg i.p.) or saline injection. Cardiac function of both the control and LPS-pretreated groups was deteriorated during reperfusion after global ischemia (Figure 1).

The post-ischemic aortic flow, coronary flow, cardiac output, heart rate, left ventricular developed pressure, and its first derivatives (±d*p*/d*t*_max_) were decreased (Figure 1A–G), while left ventricular end-diastolic pressure was increased (Figure 1H) compared to the pre-ischemic values. However, post-ischemic decline of aortic flow, cardiac output, left ventricular developed pressure, and +d*p*/d*t*_max_ was significantly improved by LPS pretreatment (Figure 1A,C,E,F). Coronary flow, heart rate, −d*p*/d*t*_max_, and left ventricular end-diastolic pressure were not affected significantly by low-dose LPS pretreatment after the ischemia (Figure 1B,D,G,H). There was no difference in cardiac performance before the ischemia between control and LPS-pretreated animals (Figure 1). Post-ischemic LDH release was significantly reduced by low-dose LPS treatment (Table 1). There was no difference in animal weight, heart wet weight, and basal heart rate between the experimental groups (Table 1).

### 2.2. LPS Pretreatment Enhances Cardiac 3-Nitrotyrosine Formation

**Figure 2:**
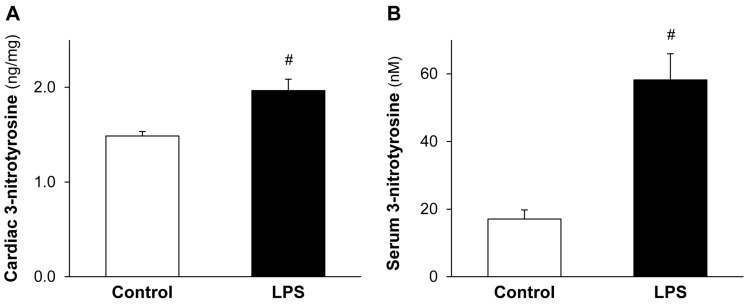
LPS pretreatment enhances cardiac and serum 3-nitrotyrosine formation. Figure shows cardiac (**A**) and serum (**B**) 3-nitrotyrosine levels 24 h after in vivo treatment of Wistar rats with 0.5 mg/kg lipopolysaccharide (LPS). Values are expressed as mean ± S.E.M. (*n* = 7–9). *^#^ p* < 0.05 vs control, unpaired *t*-test.

To assess the delayed effect of cardioprotective LPS on ONOO^−^ formation, the level of cardiac and serum 3-nitrotyrosine, a maker of ONOO^−^ was measured. A low dose of LPS significantly enhanced both the formation of cardiac (Figure 2A) and serum (Figure 2B) 3-nitrotyrosine 24 h after the in vivo administration of LPS.

### 2.3. LPS Pretreatment Leads to Increased Level of Cardiac NO and O_2_^•−^

**Figure 3:**
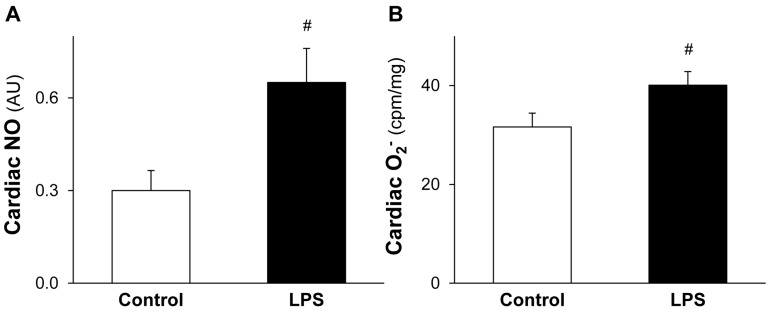
LPS pretreatment leads to increased level of cardiac NO and O_2_^•−^. Figure shows cardiac peroxynitrite precursor nitric oxide (NO) (**A**) and superoxide (O_2_^•^^−^) (**B**) levels 24 h after in vivo 0.5 mg/kg lipopolysaccharide (LPS) treatment. Values are expressed as mean ± S.E.M. (*n* = 7–12). *^#^ p* < 0.05 vs. control, unpaired *t*-test.

In order to elucidate the source of enhanced cardiac ONOO^−^ formation induced by low-dose LPS, NO, and O_2_^•−^ the precursors of ONOO^−^ were measured. The cardiac levels of both NO (Figure 3A) and O_2_^•^^−^ (Figure 3B) were significantly increased in LPS-pretreated hearts.

### 2.4. NOS and XOR Enzymes Contribute to Elevated NO and O_2_^•−^ Production Induced by LPS

**Figure 4:**
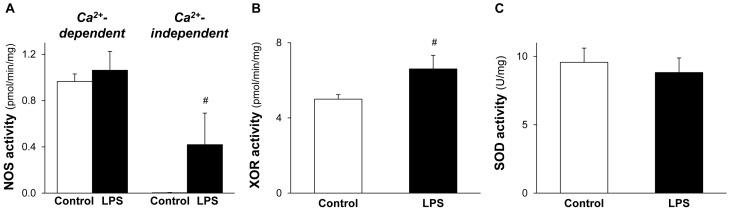
NOS and XOR enzymes contribute to elevated NO and O_2_^•−^ production induced by lipopolysaccharide (LPS). Figure shows cardiac NO synthases (NOS) (**A**), xanthine oxidoreductase (XOR) (**B**), and superoxide dismutase (SOD) (**C**) activities 24 h after in vivo 0.5 mg/kg LPS treatment. Values are expressed as mean ± S.E.M. (*n* = 7–12). *^#^ p* < 0.05 vs. control, unpaired *t*-test.

To reveal the possible source of increased cardiac NO and O_2_^•−^ levels induced by low-dose LPS, activity of NOS and XOR enzymes were measured. The activities of Ca^2+^-independent NOS and XOR were significantly increased in LPS-pretreated hearts without affecting the Ca^2+^-dependent-NOS activity (Figure 4A,B). SOD activity was not changed in response to LPS pretreatment (Figure 4C).

### 2.5. LPS Pretreatment Results in Enhanced Phosphorylation of STAT3

**Figure 5:**
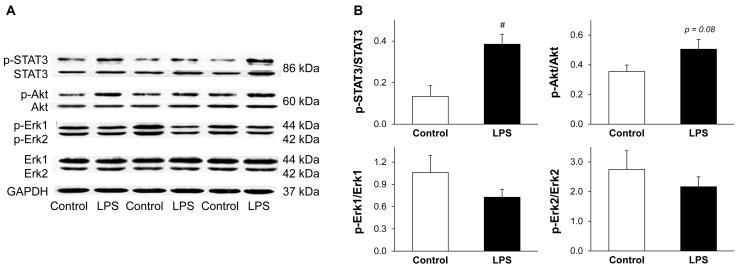
LPS pretreatment results in enhanced phosphorylation of STAT3. Figure shows representative images (**A**) and quantification (**B**) of western blots of possible cardiac pathways in LPS-induced delayed preconditioning. Analysis was performed 24 h after in vivo 0.5 mg/kg low-dose lipopolysaccharide (LPS) treatment. Values are expressed as mean ± S.E.M. (*n* = 7 in each groups). *^#^ p* < 0.05 vs. control, unpaired *t*-test. p-STAT3: phospho(Tyr705)-STAT3, p-Akt: phospho(Ser473)-Akt, p-Erk1/2: phospho(Thr202/204)-Erk1/2, GAPDH: glyceraldehyde 3-phosphate dehydrogenase.

In order to elucidate the possible downstream targets of low-dose LPS, the activations of Akt, Erk, and STAT3 were investigated 24 h after LPS pretreatment. Low-dose LPS significantly enhanced cardiac STAT3 phosphorylation and non-significantly increased Akt phosphorylation without affecting phosphorylation of Erk1/2 (Figure 5). Total STAT3 (both phosphorylated and non-phosphorylated forms) was increased by approximately 20% due to LPS pretreatment (*p* = 0.044) (Figure 5A).

## 3. Discussion

In our present study, we showed that low-dose LPS pretreatment induces late PreC by improving post-ischemic cardiac function and LDH release in isolated rat hearts. Moreover, we demonstrated for the first time in the literature that low-dose LPS pretreatment enhanced cardiac and serum 3-nitrotyrosine, a marker of ONOO^−^ formation. The precursors of ONOO^−^, NO and O_2_^•−^, were also increased in the heart as a result of LPS pretreatment. Our work revealed that enhanced Ca^2+^-independent NOS and XOR activities contribute to elevated levels of cardiac NO and O_2_^•−^. In addition, we also demonstrated an enhanced delayed phosphorylation of STAT3 after low-dose LPS pretreatment.

Ischemic PreC is a widely used method to protect the heart against ischemia-reperfusion injury [17,19]; however, the approach is invasive so it is limited to use as a preventive intervention in daily life. Pharmacological preconditioning may confer significant benefits thereby having a great potential in the clinical field including prevention of cardiovascular diseases. We demonstrated that in vivo low-dose LPS injection ameliorated post-ischemic cardiac function and LDH release in isolated rat hearts subjected to ischemia-reperfusion 24 h after the treatment. Our results are in accordance with literature data showing that endotoxin exerts late PreC by improving post-ischemic cardiac recovery [7,9,11]. Nevertheless, the molecular mechanism of endotoxin-induced delayed PreC is barely investigated. Since peroxynitrite has emerged as a potential mediator of cardioprotection [13,20,21], we focused on LPS-induced delayed cardiac ONOO^−^ formation in our present study.

Although enhanced peroxynitrite formation contributes to the pathophysiology of cardiovascular diseases by inducing oxidative- and nitrative stress [22], Lefer et al. has demonstrated that peroxynitrite inhibits leukocyte-endothelial cell interaction, which improves post-ischemic myocardial function [23]. Moreover, several further studies have shown that enhanced formation of cardiac ONOO^−^ plays a role in the early phase [12,13] as well as the late phase of ischemia-induced delayed PreC [14]. In the LPS-induced late PreC of the brain, ONOO^−^ has emerged as an early mediator [24]. We showed that low-dose LPS pretreatment enhances cardiac 3-nitrotyrosine, a marker of ONOO^−^, thereby indicating a possible role for ONOO^−^ in endotoxin-induced late PreC.

Peroxynitrite arises from the non-enzymatic reaction of NO with O_2_^•−^. In order to elucidate the source of enhanced cardiac ONOO^−^ formation induced by LPS, here we measured both precursors and found that cardiac levels of both NO and O_2_^•−^ were increased in low-dose LPS-pretreated hearts. NO has been already implicated as a mediator of the endotoxin-induced cardiac late PreC [8,9,10,25,26] and our finding is consistent with these studies. NO can be produced by three isoforms of NO syntheses: the Ca^2+^-independent inducible NOS (iNOS) and the Ca^2+^-dependent endothelial and neuronal NOS. Several studies have demonstrated the role of iNOS in delayed ischemic PreC [8,9,27]. It has been also reported that iNOS mediates endotoxin-induced late PreC as well [8,9,10,28,29]. Our findings support these data since low-dose LPS pretreatment increased the activity of Ca^2+^-independent NOS without affecting the Ca^2+^-dependent isoforms in our present study.

Besides the role of NO, indirect evidence suggested that O_2_^•−^ may also be involved in endotoxin-induced late cardioprotection [7,11]; however, our study provided direct data that cardiac level of O_2_^•−^ is increased after a low-dose LPS pretreatment. O_2_^•−^ is produced by enzymatic and non-enzymatic processes. XOR is a prominent enzymatic source for O_2_^•−^ [30], and in addition, XOR has been reported to contribute to post-ischemic preservation of left ventricular developed pressure in early ischemic PreC [31]. The potential role of XOR in endotoxin-induced delayed PreC is not known. It was reported in endotoxemic animal models that high-dose LPS induces XOR in the heart [32] and in the lung [33] 6 h or 24 h after the treatment, respectively. However, our study has revealed that cardioprotective low-dose LPS pretreatment 24 h later increases the activity of XOR enzyme in the heart. This finding suggests that XOR contributes to LPS-induced delayed PreC by producing O_2_^•−^.

We also investigated the activation of RISK and SAFE pathways in our present study to further explore possible downstream mechanisms of LPS-induced late PreC. These cardioprotective signalling pathways are barely investigated in endotoxin-induced late PreC. Non-LPS induced activation of Akt (member of RISK) and STAT3 (member of SAFE) before a lethal injury was shown to confer cardioprotection [34,35]. Ischemic PreC stimulus itself (i.e., brief ischemia-reperfusion cycles) increases cardiac Akt phosphorylation before a test ischemia [36] and in the present study, we showed that LPS pretreatment also non-significantly increased Akt phosphorylation before the test ischemia. Post-ischemic activation of Akt in response to LPS pretreatment was demonstrated by Ha et al. [18]; however, data on pre-ischemic Akt activation is lacking in that study. Although the transcription factor STAT3 has been implicated in late ischemic PreC [37,38], its role in endotoxin-induced delayed PreC has not yet been investigated. This is the first demonstration that low-dose LPS enhances the phosphorylation of cardiac STAT3 24 h after the treatment, thereby suggesting that activation of STAT3 before the ischemia may play a role in endotoxin-induced late PreC. Our hypothesis is supported by a finding that hydrogen peroxide-induced PreC stimulus itself activates STAT3 in PC12 cells before a lethal injury, which contributes to protection [39]. We also showed increased total STAT3 level in response to LPS pretreatment, which is consistent with literature data [40,41] and may indicate protein-level changes.

Although specific clinical translational goals were beyond the scope of the present experimental work, our findings may contribute to progress the current science on several fields. First, the role of peroxynitrite in cardioprotection and the interplay between moderate oxidative stress and cardioprotective pathways are still unclear. Our results indicate that enhanced peroxynitrite formation and STAT3 phosphorylation (together or separately) may play a role in LPS-induced cardioprotection. Based on literature data, it is feasible to suggest that peroxynitrite itself leads to STAT3 phosphorylation [42]. This finding may facilitate further proof-of-concept studies to elucidate the exact interplay between peroxynitrite and STAT3 in cardiomyocytes during adaptive processes. Second, our results may strengthen the concept that activation of STAT3 transcription factor seems to be a significant step in cardioprotection [43], which may support the importance of developing novel STAT3 activators or modulators that can be tested for their potential cardioprotective effects. Third, as we also showed that LPS pretreatment has a great preventive potential, our findings may promote new research to find clinically applicable analog molecules of LPS to induce pharmacological preconditioning.

In conclusion, low-dose LPS pretreatment induces pharmacological late PreC and enhances cardiac ONOO^−^ formation 24 h after the treatment by stimulating cardiac NO and O_2_^•−^ production through Ca^2+^-independent NOS and XOR enzymes. Activation of STAT3 before a lethal ischemia may play a role in the beneficial effect of endotoxin-induced delayed PreC.

## 4. Materials and Methods

Male Wistar rats (250–350 g) were used in the present study. The study conforms to the ‘Guide for the care and use of laboratory animals’ published by the US National Institutes of Health (NIH publication No. 85–23, revised 1996) and was approved by local ethics committees. The animals were kept at 12/12-hour light/dark cycle and had free access to standard laboratory chow and drinking water.

### 4.1. Materials

Bovine serum albumin (BSA), lipopolysaccharid from *Salmonella enterica* serotype *typhimurium* (#L-7261), HEPES, dithiothreitol, trypsin inhibitor, leupeptin, aprotinin, phenylmethylsulfonyl fluoride (PMSF), lucigenin, l-[^14^C]arginine, EGTA, *N*^G^-monomethyl-l-arginine, l-citrulline, pterin and methylene blue, protease inhibitor cocktail (#8340) were purchased from Sigma Aldrich (Saint Louis, MO, USA). Sucrose, Na_2_EDTA, and FeSO_4_·7H_2_O were from Reanal (Budapest, Hungary). *N*-methyl-d-glucamine-dithiocarbamate (MGD) was synthetized by Fülöp F (Department of Pharmaceutical Chemistry, Faculty of Pharmacy, Szeged, Hungary). BCA Protein Assay Kit was from Pierce (Rockford, IL, USA). Saline was from TEVA (Petah Tikva, Israel). Lactate dehydrogenase (LDH)-P kit was purchased from Diagnosticum (Budapest, Hungary). Peroxynitrite marker 3-nitrotyrosine enzyme-linked immunosorbent assay (ELISA) was from Cayman Chemical (Ann Arbor, MI, USA). Superoxide dismutase (SOD) assay was from Randox Laboratories (Crumlin, UK). Western blotting reagents were from Bio-Rad (Hercules, CA, USA). Radioimmunoprecipitation assay (RIPA) buffer and primary antibodies were purchased from Cell Signaling Technology (Danvers, MA, USA): anti-phospho(Tyr705)-STAT3 (#9145), anti-phospho(Ser473)-Akt (#9271), anti-phospho(Thr202/204)-Erk1/2 (#9101), anti-total STAT3 (#4904), anti-total Akt (#9272), anti-total ERK (#9102), anti-glyceraldehyde 3-phosphate dehydrogenase (GAPDH, #2118). HRP-conjugated secondary antibody was from Dako Corporation (Santa Barbara, CA, USA). Price Western blotting Detection Reagent was from Amersham (Buckinghamshire, UK).

### 4.2. Experimental Design and Isolated Heart Perfusion

Rats were treated intraperitoneally (i.p.) with saline or low-dose 0.5 mg/kg LPS from *Salmonella enterica* serotype *typhimurium*. Twenty four hours after LPS treatment, rats were anesthetized with diethyl ether and were given 500 U·kg^−1^ heparin intravenously. Hearts were then isolated and perfused according to Langendorff for 5 min at 37 °C with Krebs-Henseleit buffer containing NaCl 118 mM, NaHCO_3_ 25 mM, KCl 4.3 mM, CaCl_2_ 2.4 mM, KH_2_PO_4_, 1.2 mM, MgSO_4_ 1.2 mM, glucose 11 mM, gassed with 95% O_2_ and 5% CO_2_ [44,45]. Then the perfusion system was switched to working mode according to Neely with recirculating buffer [44,46]. Hydrostatic preload and afterload were kept constant at 1.7 kPa and 9.8 kPa, respectively throughout the experiments. Hearts were subjected to 10 min equilibration period followed by 30 min normothermic global ischemia and 20 min reperfusion (*n* = 6–7). Before ischemia and during reperfusion cardiac functional parameters including heart rate, coronary flow, aortic flow, left ventricular developed pressure and its first derivatives (±d*p*/d*t*_max_), and left ventricular end-diastolic pressure were measured. To estimate the severity of cellular damage in the heart, the activity of LDH was measured from coronary effluents (collected during the first 5 min of reperfusion) using a LDH-P kit (*n* = 3–4). The enzyme activity (U/mL) measured in an effluent was multiplied with the corresponding coronary flow (mL/min) to give LDH release expressed as U/min.

In separate experiments, hearts were harvested at the end of a 5-min Langendorff perfusion for biochemical analyses. After removing atria, ventricles were used freshly or were rapidly freeze-clamped, powdered with a pestle and mortar in liquid nitrogen, and stored in cryovials at −80 °C until further analysis.

### 4.3. Assessment of Cardiac and Serum ONOO^−^ Formation

To estimate cardiac and serum ONOO^−^ formation, free 3-nitrotyrosine, a marker of peroxynitrite was measured by ELISA (*n* = 7–9) as described [20,44].

Heart samples were homogenized in a buffer containing HEPES (10 mM), sucrose (0.32 M), Na_2_EDTA (0.1 mM), dithiothreitol (1.0 mM), trypsin inhibitor (10 mg/mL), leupeptin (10 mg/mL), aprotinin (2 mg/mL), and PMSF (125 µg/mL) at pH 7.4. The crude homogenates were centrifuged at 10,000× *g* for 10 min at 4 °C, and supernatants were then used for 3-nitrotyrosine quantification. Serum samples (210 µL) were mixed with four volumes 4 °C ethanol, and centrifuged at 3000× *g* for 10 min at 4 °C. Supernatants were evaporated under a flow of nitrogen and redissolved in 105 µL ultra-pure water.

According to the manufacturer’s instructions, supernatants from the heart homogenates and redissolved supernatants from the serum samples were incubated overnight at 4 °C with nitrotyrosine acetylcholinesterase tracer and anti-nitrotyrosine rabbit IgG in microplates precoated with mouse anti-rabbit IgG. Ellman’s reagent was then used for development. Free nitrotyrosine content was normalized to protein content of cardiac homogenates and expressed as ng/mg protein. Serum nitrotyrosine concentration was expressed as nmol/L.

### 4.4. Measurement of Cardiac NO and O_2_^•−^ Levels

Since ONOO^−^ is formed as a result of the reaction of NO and O_2_^•−^, the cardiac level of these intermediates were also measured in the present study.

The level of NO in ventricular tissue was measured by electron paramagnetic resonance (EPR) spectroscopy as described [47,48]. Hearts were loaded with the freshly prepared NO-specific spin trap Fe^2+^(MGD)_2_. The spin trap (175 mg MGD and 50 mg FeSO_4_ dissolved in 6 mL distilled water and pH set to 7.4) was infused for 5 min into the aorta during Langendorff perfusion at a rate of 1 mL/min. At the end of the infusion of Fe^2+^(MGD)_2_, myocardial tissue samples were collected, minced, and pushed into the bottom of quartz EPR tubes and frozen carefully in liquid nitrogen. EPR spectra of NO-Fe^2+^-(MGD)_2_ adducts were recorded with an EPR spectrometer (model ECS106, Bruker; Rheinstetten, Germany) and analyzed for NO signal intensity as described [49,50].

Cardiac O_2_^•−^ production was assessed by lucigenin-enhanced chemiluminescence as described earlier [44,51]. The apex of the heart was cut into small pieces and placed in Krebs-Henseleit buffer containing 10 μmol/L lucigenin and 10 mmol/L HEPES-NaOH (1 mL, pH 7.4). Luminescence was measured using a liquid scintillation counter (Tri-Carb 2100TR, Packard Instrument Company, Meriden, CT, USA) as described [21,30].

### 4.5. Measurement of Cardiac NO Synthases (NOS), Xanthine Oxidoreductase (XOR), and SOD Activities

Powdered frozen ventricular tissue was homogenized in 4 volumes (NOS, XOR) or 10 volumes (SOD) of ice-cold homogenization buffer (composition is same as described above for ONOO^−^ measurement) with an Ultra-Turrex disperser using three 20-s strokes. The homogenate was centrifuged (1000× *g* for 10 min) at 4 °C and the supernatant was kept on ice for immediate assays of enzyme activities.

To determine enzymatic NO production in the hearts, Ca^2+^-dependent and -independent NOS activities were assessed as described [44,51]. The conversion of l-[^14^C]arginine to l-[^14^C]citrulline was measured in supernatants with or without EGTA (1 mM) or EGTA plus *N*^G^-monomethyl-l-arginine (1 mM) to estimate Ca^2+^-dependent and -independent NOS activities, respectively. NOS activities were expressed in pmol/min/mg protein.

Since XOR is a major source of superoxide in the rat heart [52], the activity of XOR was determined in supernatants. A fluorometric kinetic assay was performed as described previously [44,53], by measuring the conversion of pterine to isoxanthopterine in the presence as well as in the absence of methylene blue.

SOD activity was measured by a spectrophotometric assay based on the inhibition of superoxide-induced formazan dye formation [51].

### 4.6. Investigation of Akt, Erk, and STAT3 Activation with Western Blot

To assess the activation of Akt, Erk and STAT3, the phosphorylation rate of these proteins was determined by Western blot as described earlier [54,55] with some modifications. Briefly, powdered ventricular tissue samples were homogenized in RIPA buffer supplemented with protease inhibitors using an ultrasonicator (UP100H Hielscher, Teltow, Germany). The homogenates were spun at 15,000× g (30 min, 4 °C). The protein concentrations in each supernatant were determined by the BCA assay. Reduced and denaturized samples (25 μg protein) were loaded on 10% polyacrylamide gel, and proteins were separated by standard SDS-PAGE (90 V, 1.5 h) followed by wet-transfer onto nitrocellulose membrane (20% methanol, 35 V, 2 h). Membranes blocked in 5% *w*/*v* BSA (1 h, room temperature) were incubated with primary antibodies generated against the following antigens: phospho(Ser473)-Akt (1:500), total Akt (1:2000); phospho(Thr202/Tyr204)-Erk1/Erk2 (1:2000), total Erk1/Erk2 1:1000; phospho(Tyr705)-STAT3 (1:1000), total STAT3 (1:2000) (overnight, 4 °C, 5% BSA); or GAPDH (1:10,000; 1 h, room temperature, 1% milk). Then the membranes were incubated with a HRP-conjugated secondary antibody (1:5000 or 1:20,000 for GAPDH; 1 h, room temperature, 1% milk). Enhanced chemiluminescence kit was used to develop the membranes.

### 4.7. Statistical Analysis

Data were expressed as mean ± S.E.M. and analyzed with unpaired *t*-test or two-way analysis of variance (ANOVA) as appropriate. If a difference was established in ANOVA, Fisher’s Least Significant Difference (LSD) *post hoc* test was applied. Differences were considered significant at *p* < 0.05.

## Figures and Tables

**Table 1:** Morphological parameters and LDH release.

	Control	LPS
Animal weight (g)	307 ± 5	301 ± 5
Heart wet weight (mg)	928 ± 25	927 ± 28
Basal heart rate (bpm)	299 ± 7	295 ± 11
LDH release (U/min)		
*Before ischemia*	1.1 ± 0.2	1.5 ± 0.3
*After ischemia*	5.8 ± 0.7 *	2.3 ± 0.4 ^#^

Values are expressed as mean ± S.E.M (*n* = 3–7 in each groups). * p < 0.05 vs before ischemia, *^#^ p* < 0.05 vs control, two-way ANOVA.
